# Traumatic childhood experiences and personality functioning: effect of body connection in a cross-sectional German and Chilean sample

**DOI:** 10.1186/s40479-024-00266-z

**Published:** 2024-08-27

**Authors:** Katja Bertsch, Isabelle Göhre, Marianne Cottin, Max Zettl, Carolin Wienrich, Sarah N. Back

**Affiliations:** 1 Department of Psychology, Julius-Maximilians-Universität Würzburg, Würzburg, Germany; 2 German Center for Mental Health (DZPG), Partner Site Munich, Munich, Germany; 3 Department of Psychology, Ludwig-Maximilians-Universität München, München, Germany; 4 Escuela de Psicología, Universidad Adolfo Ibañez, Santiago, Chile; 5 Institute for Psychosocial Prevention, University Hospital Heidelberg, University of Heidelberg, Heidelberg, Germany; 6 Institut Human-Computer-Media, Julius-Maximilians-Universität Würzburg, Würzburg, Germany

**Keywords:** Childhood trauma, Personality functioning, Personality disorder, Body dissociation, Embodiment

## Abstract

**Background:**

Traumatic childhood experiences are a major risk factor for developing mental disorders later in life. Over the past decade, researchers have begun to investigate the role of early trauma in impairments in personality functioning following the introduction of the Alternative Model of Personality Disorders in Section III of the Diagnostic and Statistical Manual for Mental Disorders 5. Although first studies were able to empirically demonstrate a significant link between early trauma and impairments in personality functioning, only little is known about the underlying mechanisms. One possible mechanism is body connection due to its involvement in self-regulatory processes and its link to both early trauma and personality (dys)functioning.

**Objective:**

In the current study, we investigated whether body connection, which encompasses the awareness, integration, and utilization of one’s own bodily signals, mediates the relationship between childhood trauma and personality functioning.

**Participants and setting:**

A total of 1,313 adult participants recruited in Germany and Chile anonymously provided self-report data in an online survey.

**Methods:**

Self-report data included the short form of the Childhood Trauma Questionnaire (CTQ-SF), the Scale of Body Connection (SBC), and the brief form of the Levels of Personality Functioning Scale (LPFS-BF 2.0) as well as demographic data (age, sex, education, clinical diagnoses).

**Results:**

Traumatic childhood experiences explained 27.2% of the variance in impairments in personality functioning. Interestingly, 60.5% of this effect was explained by body connection, particularly body dissociation. Additional exploratory analyses revealed that body dissociation and, to a much lesser extent, body awareness, accounted for 64.41% of the variance in self functioning and 55.75% of the variance in interpersonal functioning explained by childhood trauma.

**Conclusion:**

Body connection appears to be an important mediator in the association between early trauma and impaired personality functioning, underscoring the need for interventions specifically targeting the avoidance and ignorance of signals from one’s own body in individuals with traumatic childhood trauma.

**Supplementary Information:**

The online version contains supplementary material available at 10.1186/s40479-024-00266-z.

## Introduction

More than half of children worldwide are exposed to some form childhood trauma [17]. Childhood trauma encompasses any form of aversive interpersonal experience before the age of 18 years, including physical, sexual, and/or emotional abuse, as well as physical or emotional neglect [8]. It is considered the most important single risk factor for the development of a mental disorder later in life [30].

Negative consequences throughout one’s lifetime may be explained by effects of childhood trauma on the development of regulatory skills, which play a crucial part in personality development until adulthood [20, 31]. Motivated by the relatively new concept “Levels of Personality Functioning” of the Alternative Model of Personality Disorders (AMPD) in Section III of the Diagnostic and Statistical Manual for Mental Disorders 5 (DSM-5; [1]), the relationship between childhood trauma and personality functioning has recently garnered much attention. Within the AMPD, impairments in *self functioning* hierarchically map to deficits in identity (e.g., unclear boundaries between oneself and others, unstable self-worth, and deficits in emotion recognition and regulation) and self-direction (e.g., incoherent or unstable personal goals, evaluation standards, and self-reflection). Impairments in *interpersonal functioning* encompass deficits in empathy (e.g., difficulties comprehending and appreciating other peoples’ experiences, perspectives, and the effect of one’s own behavior on others) and intimate relationships (e.g., the desire and capacity for intimate, stable, and mutual interpersonal relationships; [1]). The dimensional conceptualization of levels of personality functioning according to the AMPD integrates various processes that have recently been conceptualized as central mediators in the relationship between traumatic childhood experiences and psychopathology. These mediators, such as emotion dysregulation and deficits in social information processing, can play a role across diagnostic entities or within specific disorders (for review, see [31]).

Indeed, there is accumulating evidence from cross-sectional studies supporting a diathesis-stress model of personality functioning. According to this model, impairments in personality functioning may represent a risk process across diagnostic entities, associated with increased vulnerability to psychopathology in general following early trauma [12, 14, 18, 21]. Cross-sectional studies have revealed that individuals with a higher self-reported history of traumatic childhood experiences exhibit more severe impairments in self and interpersonal functioning (e.g., [5]). Nevertheless, much remains unknown about the underlying processes. Identifying potential mechanisms is of urgent relevance for the development of empirically informed treatments, which are still lacking for dimensionally assessed impairments in self and interpersonal functioning.

Recently, deficits in *body connection*, specifically disconnection between bodily and mental processes, have been proposed and studied as one such mediating process between traumatic childhood experiences and impairments in self and interpersonal functioning in individuals with borderline personality disorder (BPD) ([41]; for reviews on this topic, see [4, 26]). The importance of connecting bodily experiences with cognitive, emotional, and behavioral processes has been emphasized by earlier embodiment theories, which propose that humans think, feel, and behave in an “embodied” manner [11]. According to a theory proposed by Damasio [11], emotional experiences are embodied as somatic markers. These markers facilitate intuitive decision-making and guide interpersonal behavior (both approach and avoidance) based on past experiences that are similar in nature. These somatic markers consist of bodily signals and/or physiological changes that are linked to situation-specific cognitions, emotions, and behavioral scripts. They are thought to provide behavioral guidance [11]. Neuroanatomically, bodily awareness and processing related to self and others have been theorized to converge within the insular cortex, which is considered the center of self-awareness [9]. Indeed, the anterior insular cortex is not only implicated in the conscious perception of bodily sensations, but also in emotional experiences [10]. It is thought to form representations of the self and to integrate external social information [9]. Theories have emphasized the crucial role of negative early experiences in the development of severe personality disorders, such as BPD. For instance, Linehan [25] proposed that particularly the invalidation of negative early experiences by primary caregivers hinders learning to recognize, listen to, and utilize own (emotional) reactions as behavioral guidance. Growing up in an abusive and invaliding environment may instead promote attentional focus on external signals of potential threat, hindering the development of self-regulatory skills, particularly the regulation of own emotions and behavioral impulses. Consequently, this may interfere with development of a stable and coherent identity and self-worth, and interpersonal functioning. Interestingly, Löffler and colleagues [26] have added interoceptive processes, including the ability to perceive and utilize one’s own bodily signals, as a central mediator to this model (also see, [4]). The avoidance and/or ignorance of and dissociation from one’s own bodily sensations could serve as a way to cope with negative emotions associated with past traumatic events [41].

So far, there is some empirical evidence for the model of Löffler et al. [26] from studies involving healthy and clinical populations. Reduced body connection, which encompasses deficits in the awareness, attention, and perception of bodily signals, as well as the avoidance and/or ignorance of them (oftentimes summarized as “body dissociation”), has been linked not only to early trauma but also impairments in personality functioning. For example, healthy individuals with traumatic childhood experiences were found to exhibit an attenuated increase in body perception following an acute social stressor [39] or a pharmacological stress induction [43]. Additionally, the association between early trauma and emotion dysregulation was fully mediated by body dissociation in individuals with BPD [41] as well as individuals with different trauma-related disorders, including major depression (MD), posttraumatic stress disorder (PTSD), and somatic symptom disorder (SSD), along with healthy individuals [42].

However, whether body connection also mediates the association between childhood trauma and personality functioning according to the DSM-5 AMPD remains unclear. The current study aimed to close this gap by collecting self-report data on childhood trauma, body connection, and personality functioning from a large, heterogeneous sample, including individuals from two different countries, namely, Germany and Chile. Based on previous findings, we hypothesized that (1) childhood trauma would be significantly related to impairments in personality functioning and (2) that body connection would significantly mediate this association. Additionally, we (3) explored whether this mediating effect was specific to impairments in self and/or interpersonal functioning. Since we were explicitly interested in the mediating role of body connection as proposed in the model by Löffler and colleagues [26] and suggested by results in patients with BPD, we focused on mediation models.

## Methods

### Participants

The online survey study was conceptualized in a manner to reach a heterogenous sample encompassing various states of mental health and personality functioning, as well as diverse countries of origin. Therefore, participants were recruited in Germany and Chile. In Germany, we used PsyWeb (https://psyweb.uni-muenster.de/), a scientific survey panel with the explicit goal to reach a diverse sample from the community. Since such a panel is not available in Chile, we had to rely on online announcements, advertisements on social media and universities as well as from psychiatric hospital websites.

Following these strategies, data from *N* = 1,313 individuals were gathered in total. *N* = 800 individuals were recruited in Germany and *N* = 529 individuals in Chile. The Chilean sample comprised *N* = 233 participants from the community and *N* = 296 recruited from psychiatry hospital websites. Individuals of this latter group had a self-reported diagnosis of a personality disorder at any point in their lives and received psychological and/or psychiatric treatment at the time of the survey. General inclusion criteria consisted of being at least 18 years of age, having sufficient proficiency in either German or Spanish language, and providing documented informed consent.

Data were collected anonymously using online self-report questionnaires on the platform “SoSciSurvey” between February and April 2021. As compensation and incentive, participants had the opportunity to participate in a lottery for ten €25-Amazon gift cards. For those who opted to participate in this voluntary lottery, email addresses were requested and stored separately from all other data to ensure anonymity. Email addresses were solely used to contact the lottery winners to distribute the gift cards and were deleted thereafter. It was not possible to link the self-report questionnaire data with the email addresses at any point during or after the study.

The study was designed in accordance with the ethical principles of the Declaration of Helsinki and was reviewed and approved by the ethics committees of the Department of Psychology, Ludwig-Maximilians-University, Munich, Germany, and of the Universidad de Chile, Santiago de Chile, Chile.

### Self-report questionnaires

#### Childhood Trauma Questionnaire - Short Form (CTQ-SF)

The Childhood Trauma Questionnaire - Short Form (CTQ-SF; [8]) is the most widely used self-report questionnaire for assessing retrospective childhood trauma. It encompasses 28-items [7], which evaluates self-reported emotional abuse, sexual abuse, physical abuse, emotional neglect, and physical neglect before the age of 18 within five respective subscales (5 items/subscale plus 3 items assessing bagatellization, which were not included in the analyses), using a five-point Likert-scale (ranging from 1 = *never true* to 5 = *very often true*). The CTQ-SF has demonstrated good convergent validity [8], factor validity, reliability [40], and measurement invariance [8] across diverse populations. In the current study, we utilized validated German [47] and Spanish [6] versions of the CTQ-SF. A Cronbach’s alpha of α = 0.94 for the total sample as well as α = 0.94 for the German sample and α = 0.94 for the Chilean sample indicated excellent reliability in the current study (excluding bagatellization items).

#### Scale of Body Connection (SBC)

The Scale of Body Connection (SBC; [35]) assessed body awareness and body dissociation over the past two months with two separate subscales. The body awareness subscale evaluates attention to and perception of bodily signals in everyday life, including bodily reactions to emotions, with 12 items. The body dissociation subscale measures the tendency to avoid and/or ignore internal bodily experiences, including feelings of detachment or disconnection from one’s own body, with eight items. Participants respond to all items on a five-point Likert scale (ranging from 0 = *not at all* to 4 = *all the time*). The average score for each subscale has been used as measures for body awareness and body dissociation in various studies and countries (see [36] for an international validation study). In the current study, we utilized a back and forth translated, unvalidated German version [41] of the English original [35], as well as the validated Spanish version [38]. For the subscale body awareness, Cronbach’s alpha indicated acceptable reliability at α = 0.83 in the whole sample as well as α = 0.82 in the German sample and α = 0.85 in the Chilean sample, in the current study. Moreover, the subscale body dissociation also displayed an acceptable internal consistency with a Cronbach’s alpha of α = 0.81 in the whole sample as well as α = 0.83 in the German sample and α = 0.79 in the Chilean sample.

#### Level of Personality Functioning Scale – Brief Form 2.0 (LPFS-BF 2.0)

The Levels of Personality Functioning Scale – Brief Form 2.0 (LPFS-BF 2.0; [46]) is a 12-item self-report measure of personality functioning according to criterion A of the Alternative Model of Personality Disorders (AMPD). Items one to six assess the dimension of self functioning, while items seven to 12 assess interpersonal functioning. Participants rate all items on a five-point Likert-scale (ranging from 0 = *very false or often false* to 3 = *very true or often true*). The sum scores for the two subscales are used as measures for self and interpersonal functioning, respectively. Higher sum scores indicate of higher impairments in personality functioning, in accordance with the DSM-5 levels of personality functioning scale (APA 2019). Latent factor structure, convergent validity, and reliability of the LPFS-BF 2.0 have been confirmed in several studies [3, 46]. In the current study, we utilized the validated German version [45] and the measurement-invariant Spanish version (Cottin et al., in preparation, as employed in [33] of the LPFS-BF 2.0. For the total scale, Cronbach’s alpha indicated good to excellent reliability at α = 0.90 in the whole sample as well as α = 0.88 in the German sample and α = 0.91 in the Chilean sample. The self functioning subscale showed a good Cronbach’s alpha of α = 0.88 in the whole sample as well as in the German (α = 0.87) and Chilean (α = 0.89) sample. For the interpersonal functioning subscale, Cronbach’s alpha indicated acceptable reliability at α = 0.79 in the whole sample as well as in the German (α = 0.75) and Chilean (α = 0.83) sample.

### Data analysis

The purpose of the study was to investigate whether body connection mediates the association between childhood trauma and impairments in personality functioning. After data inspection and quality checks, bivariate correlations among all variables of interest (i.e., CTQ-SF total score, average scores of the SBC subscales body awareness and body dissociation, as well as the LPFS-BF 2.0 sum score and the sum scores of the self and interpersonal functioning subscales) were examined using Spearman correlations. Next, a parallel mediation model was conducted to investigate the direct and indirect effects of childhood trauma (CTQ-SF total score) on personality functioning (LPFS-BF 2.0 total score) simultaneously through body awareness and body dissociation (SBC subscales) as parallel mediators. Finally, the same direct and indirect effects of childhood trauma on self functioning (LPFS-BF 2.0 subscale) and interpersonal functioning (LPFS-BF 2.0 subscale) through body awareness and body dissociation as parallel mediators were explored in two additional mediation models. Separate mediation models for the German and the Chilean sample are presented in the Online Supplementary Material.

Since visual and statistical inspection revealed a violation of the assumption of normality for all variables of interest, a bootstrapping sampling procedure was applied to robustly estimate all effects (with 10,000 bootstrapped samples), which is a non-parametric approach allowing for more accurate inferences in case of not-normally distributed data [32]. This approach is in line with official recommendations for mediation analysis as provided by Hayes [15] and MacKinnon et al. [27]. Significance at the level of α = 0.05 (two-sided) was considered significant only if zero was not included within respective confidence intervals. Control variables including age, sex, and country (Germany, Chile) were included in all mediation models to adjust for their influence. Statistical analyses were conducted in SPSS, using the process macro (version 4.2) for mediation analysis by Hayes [15].

## Results

### Descriptives

From the original *N* = 1,399 individuals who enrolled in the study, *N* = 29 participants responded at least twice as fast as the average participant based on the Relative Speed Index [24], which is a reliable indicator for meaningless data based on relative completion times, and were subsequently excluded from any further analyses. Additionally,* N* = 2 participants reported an age below 18 years, and *N* = 55 participants did not provide information on the CTQ-SF, so that they were also removed from any further analyses that were based on *N* = 1,313 individuals. Due to technical problems, demographic data from *N* = 31 Chilean participants were not saved and thus had to be excluded from respective parts of the descriptive and mediation analyses. Consequently, mediation analysis was conducted on *N* = 1,282.

Descriptive analyses for the total sample, as well as separately for the German and Chilean samples, are provided in Table 1. The sample consisted mostly of female participants (76.6% reporting female gender), with an average age of *M*_age_ = 32.87 years (*SD*_age_ = 9.76). Most participants were employed or in academic/vocational training (85%). Approximately half of the participants (42.2%) reported a current or past mental disorder.

**Table 1:** Sample description

	Total	Chile	Germany
	(*N* = 1,313)	(*N* = 519)^a^	(*N* = 794)
**Sex***, n* (%)			
Female	981 (76.52%)	379 (77.66%)	602 (75.82%)
Male	288 (22.46%)	108 (22.13%)	180 (22.67%)
Diverse	13 (1.01%)	1 (0.20%)	12 (1.51%)
**Age, ***years*			
Mean (*SD*)	32.87 (9.76)	31.37 (10.59)	33.79 (9.10)
Range	18–74	18–74	18–55
**Occupation**^b^ , *n (%)*			
Employed	613 (47.82%)	115 (23.57%)	498 (62.72%)
Student/Trainee	467 (36.43%)	255 (49.13%)	212 (26.70%)
Unemployed	202 (15.76%)	118 (22.73%)	84 (10.50%)
**Clinical History**, *n (%)*			
Mental disorder^c^	540 (42.12%)	296 (60.66%)	244 (32.02%)
No Mental disorder	742 (57.88%)	192 (39.34%)	550 (72.18%)
**Childhood Trauma**			
Mean (*SD*)	45.32 (17.04)	46.46 (17.54)	44.57 (16.67)
Range	25–123	25–120	25–123
**Personality Functioning**			
*Total score*			
Mean (*SD*)	25.47 (8.12)	23.76 (8.78)	26.59 (7.45)
Range	12–48	12–48	12–46
*Self*			
Mean (*SD*)	14.00 (4.95)	12.95 (5.19)	14.68 (4.67)
Range	6–24	6–24	6–24
*Interpersonal*			
Mean (*SD*)	11.48 (3.90)	10.81 (4.26)	11.91 (3.59)
Range	6–24	6–24	6–24
**Body Connection**			
*Body Awareness*			
Mean (*SD*)	2.40 (0.70)	2.49 (0.74)	2.35 (0.67)
Range	0–4	0–4	0–3.92
*Body Dissociation*			
Mean (*SD*)	1.22 (0.74)	1.17 (0.75)	1.26 (0.73)
Range	0–3.88	0–3.88	0–3.88

*Abbreviations*: *TCE* Traumatic Childhood Experiences measured by the sum score of the Childhood Trauma Questionnaire, *LPFS* Levels of Personality Functioning measured by the Levels of Personality Functioning Scale-Brief Version 2, *Body connection* Body awareness and dissociation measured by the Scale of Body Connection

^a^*N* = 31 participants from Chile did not indicate age, sex, occupation, or clinical status. Therefore, descriptive data was based on *N* = 488 for the respective variables

^b^Employed = actively and primarily pursuing a financially compensated job, including salaried employees, civil servants, self-employed persons, and contract-based work on an honorary basis; Student/Trainee = actively and primarily pursuing vocational or academic training, including university students and vocational trainees (pre- and postgraduate), Unemployed = not pursuing actively any financially compensating job or vocational/academic training

^c^Self-reported official record of a mental disorder diagnosed by a licensed psychotherapist or medical doctor; For the Chilean sample = diagnosis of a personality disorder at any moment of their life and a concurrent psychological or psychiatric treatment; For the German sample = diagnosis of any current mental disorder (most frequent self-reported diagnoses were affective disorders (20.20%), personality disorders (9.30%), anxiety disorders (7.30%), post-traumatic stress disorder (7.20%) and eating disorders (4.30%))

On average, participants reached a CTQ-SF total score of *M* = 45.32 (*SD* = 17.04). 19.2% of participants reported one form of traumatic experience. Two different types of traumatic experiences in childhood were reported by 15.8% of participants, three types by 14.9%, four types by 11.3%, and all five types of traumatic experiences by 7.4%, respectively. Among all five forms of traumatic childhood experiences measured by the CTQ-SF, emotional abuse was the most frequently reported (in 53.3% of the sample), followed by physical neglect (41.6%), emotional neglect (29.4%), sexual abuse (28.1%), and physical abuse (25.4%).

The average level of personality functioning in the LPFS-BF 2.0 was *M* = 25.47 (*SD* = 13.47) of the current sample equals to a T-score of 46, indicating low impairments of personality functioning on average [45].

The average score of body awareness was *M* = 2.40 (*SD* = 0.70) and the average score of body dissociation was *M* = 1.22 (*SD* = 0.74) which is comparable to previously reported data in samples from Italy, France, Netherlands, Portugal, USA, and Israel ([36]: body awareness: 2.09 ≥ *M* ≥ 2.83, 0.41 ≤ *SD* ≥ 0.84 and body dissociation: 0.79 ≤ *M* ≥ 1.43, 0.41 ≤ *SD* ≥ 0.83).

### Association between childhood trauma, body connection, and personality functioning

Correlational analyses revealed a moderate positive association between childhood trauma (CTQ-SF total score) and body dissociation (*r* = 0.449, *p* < 0.001) and a small negative correlation between childhood trauma and body awareness (*r* = -0.100, *p* < 0.01) of the SBC. Childhood trauma was also moderately positively correlated with impairments in personality functioning (*r* = 0.456, *p* < 0.001), and the same was true for the two subscales impairments in self functioning (*r* = 0.405, *p* < 0.001) as well as in interpersonal functioning (*r* = 0.436, *p* < 0.001) of the LPFS-BF 2.0. Finally, there was a strong positive correlation between impaired personality functioning and body dissociation (*r* = 0.728, *p* < 0.001) and a small negative correlation between impaired personality functioning and body awareness (*r* = -0.230, *p* < 0.001).

### Mediating effect of body connection

**Fig. 1:**
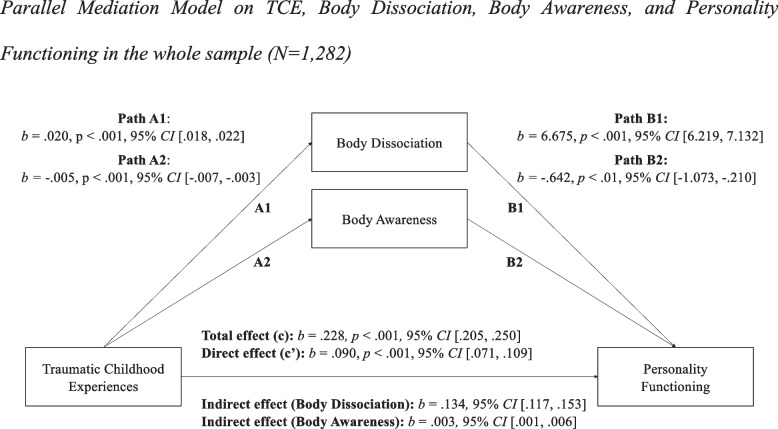
Parallel Mediation Model on TCE, Body Dissociation, Body Awareness, and Personality Functioning in the whole sample (*N* = 1,282)

Three mediation models were calculated to investigate our main research question, namely the mediating effect of body dissociation in the association between childhood trauma and impairments in personality functioning. A first model showed that body awareness and body dissociation significantly mediated the association between childhood trauma and personality functioning (see also Fig. 1): Traumatic childhood experiences explained 27% of the variance of impairments in personality functioning after adjusting for age, sex, and country (*R*^*2*^ = 0.272). Furthermore, there was a significant indirect effect of childhood trauma on impairments in personality functioning through body dissociation (*b* = 0.134, 95% *CI* [0.117, 0.153]), and, albeit to a much smaller degree, through body awareness (*b* = 0.003, 95% *CI* [0.001, 0.006]). The direct effect (c’) remained significant after the mediators were included, but its effect was substantially reduced, indicating partial mediation. The total mediating effect of body awareness and body dissociation accounted for 60.5% of the total effect of childhood trauma on impairments in personality functioning. The total model explained 58% of the variance of impairments in personality functioning (*R*^*2*^ = 0.575).

**Fig. 2:**
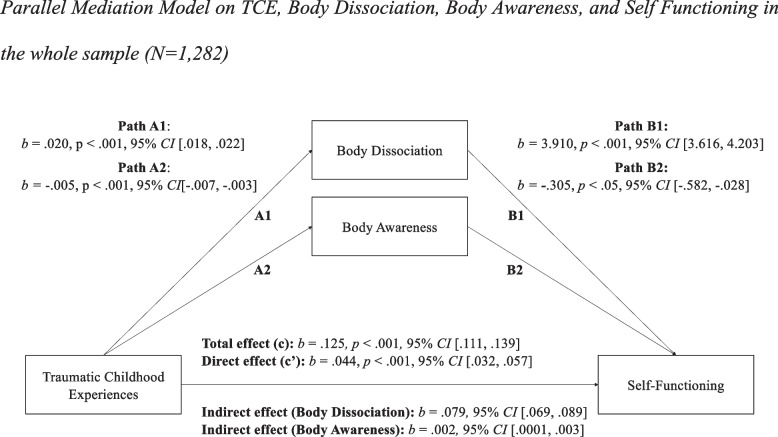
Parallel Mediation Model on TCE, Body Dissociation, Body Awareness, and Self Functioning in the whole sample (*N* = 1,282)

**Fig. 3:**
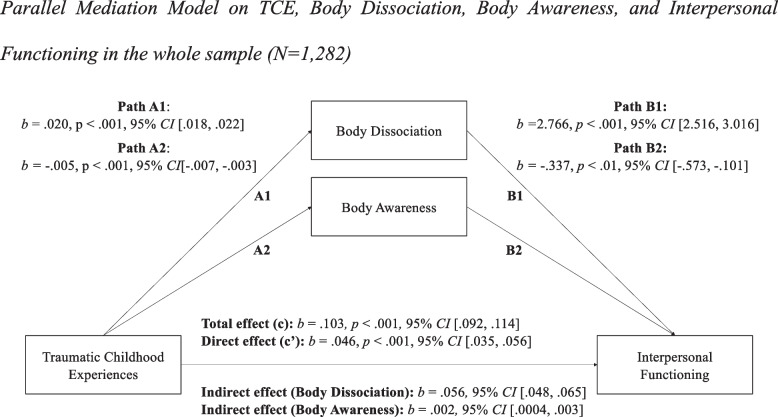
Parallel Mediation Model on TCE, Body Dissociation, Body Awareness, and Interpersonal Functioning in the whole sample (*N* = 1,282)

In a second step, we calculated two additional models to examine the mediating role of body connection on impairments in self and interpersonal functioning, separately. After adjusting for the above-described control variables, childhood trauma explained 25% of the variance in impairments in self functioning (Fig. 2) as well as 22% of the variance of impairments in interpersonal functioning (Fig. 3). There was a significant indirect effect of childhood trauma on impairments in self functioning (*b* = 0.079, 95% *CI*[0.069, 0.089]) and in interpersonal functioning (*b* = 0.056, 95% *CI*[0.048, 0.065]) through body dissociation, and, to a smaller degree, through body awareness (self: *b* = 0.002, 95% *CI*[0.0001, 0.003], interpersonal: *b* = 0.002, 95% *CI*[0.0004, 0.003]). The direct effect (c’) of childhood trauma on both self and interpersonal functioning remained significant after including the two mediators. The mediating effect of body dissociation and awareness accounted for 64.41% of the total effect of traumatic childhood experiences on self functioning, and for 55.75% of the total effect of traumatic childhood experiences on interpersonal functioning. In sum, the two models explained up to 53% in self functioning (*R*^*2*^ = 0.53) and 45% in interpersonal functioning (*R*^*2*^ = 0.45), respectively.

In a third step, we calculated the same three mediation models for the German and Chilean sample separately (see Supplementary Figures S1 to S6 for details). Looking at both samples separately, only body dissociation but not body awareness was a significant partial mediator of the relationship between childhood trauma and personality functioning, self functioning, and interpersonal functioning, while the small effect of body awareness was no longer significant in the Chilean sample. The indirect effect of body dissociation was highly comparable in both samples (*total score:* German sample: *b* = 0.135, 95% *CI*[0.113, 0.158], Chilean sample: *b* = 0.132, 95% *CI*[0.103, 0.163]; *self:* German sample: *b* = 0.083, 95% *CI*[0.071, 0.098], Chilean sample: *b* = 0.072, 95% *CI*[0.056, 0.088]; *interpersonal:* German sample: *b* = 0.051, 95% *CI*[0.041, 0.062], Chilean sample: *b* = 0.061, 95% *CI*[0.046, 0.076]).

## Discussion

In the current cross-sectional study, we examined the mediating effect of body connection on the relationship between traumatic childhood experiences and impairments in personality functioning in a heterogenous sample of individuals from Germany and Chile.

Confirming previous research (see [5] for a review) and our a priori hypothesis, traumatic childhood experiences were significantly associated with impairments in personality functioning. The current data revealed that 27% of variance in personality functioning can be attributed to childhood trauma, emphasizing their central role for mental health. Interestingly, 61% of this effect could be explained by reduced body connection, primarily through higher levels of body dissociation, and to a lesser extent, lower levels of body awareness. Our exploratory analyses suggest that this holds true for both domains of personality functioning, with body dissociation (and, to a much lesser extent, body awareness) explaining 64% of the variance in self functioning and 56% of the variance in interpersonal functioning attributed to traumatic childhood experiences. Building upon previous studies from our group [41, 42] and others [39, 43], the current data offer additional evidence for a critical role of body dissociation in the interaction between traumatic childhood experiences and impairments in personality functioning.

Our mediation analyses consistently identified body dissociation as a substantial and significant mediator in the relationship between traumatic childhood experiences and impaired personality functioning. The effect of body dissociation appeared to be much more pronounced than the nevertheless statistically significant mediating effect of body awareness, which is consistent with previous findings of Schmitz and colleagues [41, 42]. In these previous studies, it was found that body dissociation fully mediated the association between early trauma and emotion dysregulation, which is an important subdomain of self functioning, in individuals with BPD [41], PTSD, MD, SSD, and healthy controls [42].

So, what makes body dissociation so distinctive? Body dissociation encompasses the disrupted integration between bottom-up bodily and top-down mental processes. It thus goes beyond mere representation, awareness, and perception of bodily signals. Rather than that, our results suggest that the non-attendance and/or avoidance of bodily sensations, and their integration in higher-order emotional and cognitive processes, might be relevant in the development of impaired self and interpersonal functioning in the context of childhood trauma. More pronounced body dissociation is indicative of lower integration of aversive body sensations in emotional states [35] and may serve as a proxy for a reduced ability to perceive interoceptive signals (e.g., in terms of habitual disregard or non-attendance of signals from the body; [19]). For BPD, a model proposed by Löffler and colleagues [26] suggests that experiences of early trauma may result in impairments between the coupling of internal bodily signals with emotional states and decision-making. It is conceivable that internal bodily signals might fail to capture attention or be disregarded as irrelevant or even perceived as dangerous in affected individuals. Eventually, these processes may promote impairments in the development of central capacities related to self functioning, such as deficits in self-regulation and identity diffusion. Impairments in interpersonal functioning, including deficits in empathy and an inability to develop and maintain intimate relationships, might be a consequence or associated impairment (see [26]). The current findings, along with those by Schmitz et al. [42], may suggest that this model applies more generally to early trauma-associated impairments in personality functioning.

The close link between body connection and psychological (dys)function also indicates new approaches in therapy. Body-oriented therapeutic treatments have gained attention in the past decade for various mental disorders, including those with high prevalences of early trauma (see, for instance, [22] or [37]). Additionally, several studies have explored the efficacy of treatments aimed at improving interoceptive processes, including sensitivity to bodily signals. Despite the mixed results presented by meta-analyses summarizing methodologically and qualitatively very heterogenous investigations, promising outcomes were found amongst others for individuals with PTSD [16]. Many treatment programs for individuals with early trauma and/or impaired domains of personality functioning already incorporate body-oriented modules, such as body scans, mindful breathing, yoga, or others (e.g., Dialectic-Behavioral Therapy, [25]; new approaches as proposed by [23, 34].

Based on the current findings, one might suggest interventions that specifically target the integration of body signals into cognitive and emotional processes beyond their mere perception for individuals with early trauma. Such developments align with early embodiment theories rooted in the work of Damasio and colleagues (for instance, see [11]). According to these theories, bodily information can be viewed as *somatic markers* for specific emotions, thereby aiding intuitive behavior and decision-making. Furthermore, therapies in extended realities (XR, including virtual reality (VR) and augmented reality (AR)) could also be a completely new and promising approach. It has already been shown that presenting one’s own body using virtual avatars (virtual representation) can change body perception [13, 48], although effects on body connection have not been investigated, yet. In addition, further experimental research is needed to investigate whether changes in body dissociation during trauma-associated, emotionally arousing situations predict personality functioning, before causal conclusions may be drawn. With regard to psychotherapy, a positive effect of Eye Movement Desensitization and Reprocessing (EMDR), an established trauma-focused treatment, was found on the coupling in the heart-to-brain-direction in women with breast cancer and PTSD [28].

### Limitations and future directions

Despite some intriguing advantages of the current study, such as the relatively large and heterogeneous sample recruited in two different countries (and continents), some limitations need to be mentioned. First, our cross-sectional, correlational, mono-method design does not allow for causal inference. Since we followed a theoretical model [26] and had a clear a priori hypothesis, we decided to focus on medication models in the current study which may be critically regarded [29]. Other models (network or moderator analyses) might also be possible and/or the order of the variables should be inverted in future experimental and/or longitudinal studies. Secondly, despite the substantial variance explained by body connection, the partial mediation effect suggests that other processes not depicted in our model are also involved in the relationship between early trauma and impaired personality functioning which may be expected due to the complexity of both factors. Thirdly, we solely relied on self-report measures, which may limit the validity of the clinical characterization of our samples. Additionally, the retrospective reporting of childhood trauma may be influenced by recall bias and state-dependent memory. However, the CTQ demonstrates equivalent psychometric properties compared to interview-based assessments [44], therefore encouraging its validity of mapping retrospective childhood trauma. Fourthly, we cannot conclusively demonstrate that our effects on body dissociation are independent of other, more general forms of dissociation. This is particularly true, since the German version of the SBC has not been validated, yet. As inSchmitz et al. [41, 42], we used a back-and-forth translated version. In these former studies, we could also show that the effects of bodily dissociation remain robust even when levels of trait dissociation were statistically controlled for.

Lastly, we cannot conclude the possibility that other psychopathological symptoms, which are also closely related to disturbed body processing (such as eating disorders, for review see, [2]), former and/or current medication (which has not been assessed in the current study), or psychosocial aspects, may have influenced the association with personality impairments. Additionally, it is not possible to make general conclusions about causal directions between personality functioning and psychopathological symptoms, as this is beyond the scope of the current study. Although preliminary evidence suggests that personality functioning may be a transdiagnostic process of vulnerability for and/or against psychopathology [12, 14, 18, 21], causal evidence for the direction of effects is lacking. A bi-directional influence of personality functioning and psychopathological symptoms seems likely, thereby underscoring the importance of controlling for additional psychopathological symptoms, such as eating disorder symptoms [2], when examining the role of body connection on personality functioning.

## Conclusion

The findings of this large and heterogenous sample from two countries revealed body dissociation to be a significant and substantial cross-sectional mediator in the association between traumatic childhood experiences and impairments in personality functioning. Together with previous studies, these findings suggest interventions targeting the integration of body signals in higher-order cognitive and emotional processes in the treatment of individuals with early trauma as important focus of further research.

### Supplementary Information

Supplementary Material 1

## Data Availability

Data and materials supporting this study’s findings are available on request from the corresponding author.

## Data availability

No datasets were generated or analysed during the current study.
