# Supplementary material for: Traumatic childhood experiences and personality functioning: effect of body connection in a cross-sectional German and Chilean sample
Source: Borderline Personal Disord Emot Dysregul. 2024 Aug 27;11:20. doi: 10.1186/s40479-024-00266-z (PMC11348756; doi:10.1186/s40479-024-00266-z)
Supplement: Supplementary file 1 — Supplementary Material 1 [file 40479_2024_266_MOESM1_ESM.docx]

**Supplementary material**

**Figures**

**Figure S1**

*Parallel Mediation Model on TCE, Body Dissociation, Body Awareness, and Personality Functioning in the German Sample (N = 794)*

**Figure S2**

*Parallel Mediation Model on TCE, Body Dissociation, Body Awareness, and Self- Functioning in the German Sample (N = 794)*

**Figure S3**

*Parallel Mediation Model on TCE, Body Dissociation, Body Awareness, and Interpersonal Functioning in the German Sample (N = 794)*

**

**Figure S4**

*Parallel Mediation Model on TCE, Body Dissociation, Body Awareness, and Personality Functioning in the Chile Sample (N = 488)*

**Figure S5**

*Parallel Mediation Model on TCE, Body Dissociation, Body Awareness, and Self- Functioning in the Chile Sample (N = 488)*

**Figure S6**

*Parallel Mediation Model on TCE, Body Dissociation, Body Awareness, and Interpersonal Functioning in the Chile Sample (N = 488)*
